# Impact of fractional phosphate excretion on the relation of FGF23 with outcome in CKD patients

**DOI:** 10.1007/s40620-015-0178-0

**Published:** 2015-02-21

**Authors:** Anneke P. Bech, Annet Bouma-de Krijger, Arjan D. van Zuilen, Michiel L. Bots, Jan A. J. G. van den Brand, Peter J. Blankestijn, Jack F. M. Wetzels, Marc G. Vervloet

**Affiliations:** 1 Department of Nephrology, Radboud University Medical Center, PO Box 9101, 6500 HB, Nijmegen, The Netherlands; 2 Department of Nephrology, VU University Medical Center Amsterdam, Amsterdam, The Netherlands; 3 Department of Nephrology, University Medical Center Utrecht, Utrecht, The Netherlands; 4 Julius Center for Health Sciences and Primary Care, University Medical Center Utrecht, Utrecht, The Netherlands; 5 Institute for Cardiovascular Research (ICaR-VU) VU University Medical Center Amsterdam, Amsterdam, The Netherlands

**Keywords:** FGF23, Chronic kidney disease, Cardiovascular risk

## Abstract

**Background:**

Cardiovascular risk is increased in patients with chronic kidney disease (CKD). Fibroblast growth factor 23 (FGF23) has emerged as an important, independent predictor of outcome in CKD patients. High FGF23 may, however, be a reflection of renal tissue resistance to its actions, reflected by low fractional excretion of phosphate (FePi). We evaluated the modifying effect of FePi on the association between FGF23 and outcome in patients with CKD stage 3–4.

**Methods:**

An analysis was performed in a subset of 166 adult patients of two participating centers of the MASTERPLAN trial of whom urine samples at baseline were available to calculate FePi. Outcome was defined as a composite of death, renal failure (defined as need for renal replacement therapy or doubling of serum creatinine) and cardiovascular events (myocardial infarction, cerebrovascular accident, percutaneous transluminal coronary angioplasty or coronary artery bypass graft. Patients were categorized by FGF23 and FePi. A product term was added to Cox regression and RERIs were calculated.

**Results:**

Patients had a median estimated glomerular filtration rate (eGFR) of 36 ml/min/1.73 m^2^ [interquartile range (IQR) 27–44], serum phosphate 1.04 mmol/l (IQR 0.92–1.20), FGF23 140 RU/ml (IQR 81–236) and FePi 0.32 (IQR 0.25–0.44). A total of 96 events occurred during 5 years of follow up. LnFGF23 was a significant, independent predictor for the composite outcome [hazard ratio (HR) 2.13, 95 % confidence interval (CI) 1.53–2.95]. FePi did not modify the relation between FGF23 and outcome in these patients with CKD.

**Conclusions:**

Our study shows that FGF23 itself, but not its renal tissue resistance as reflected by FePi, is an important risk factor for clinical events in subjects with CKD stage 3–4.

## Introduction

Chronic kidney disease (CKD) is associated with a high risk for cardiovascular disease and mortality [1]. This increased risk is attributed to various traditional and non-traditional risk factors such as hypertension, diabetes [2], proteinuria [3], uric acid [4] and acidosis [5]. In recent years the role of disorders in calcium and phosphate metabolism has been emphasized, with studies reporting increased cardiovascular risk associations with higher serum phosphate [6–12], higher calcium [11], higher parathormone (PTH) [13], and lower vitamin D levels [14]. Most recently, focus has shifted to fibroblast growth factor 23 (FGF23), a phosphaturic hormone mainly produced by the osteocyte [15]. FGF23 is associated with cardiovascular (CV) morbidity and mortality in patients with CKD [16–18], and is an independent predictor for progression of kidney failure [16, 17, 19, 20]. Moreover, elevated FGF23 levels have been shown to be a risk factor for CV disease and mortality in the general population [18, 21, 22].

FGF23 is involved in phosphate metabolism. It inhibits expression of the sodium-phosphate transporters in the proximal tubuli of the kidneys, thus promoting renal phosphate excretion [23]. Elevated FGF23 levels in patients with CKD may partially be the result of FGF23 resistance. FGF23 resistance can be defined as a state in which the kidney and the parathyroid glands, the primary sites of FGF23 action, do not respond optimally to FGF23 by excreting less phosphate and suppressing PTH less efficiently compared to healthy persons, which drives FGF23 to increase. Classical FGF23 action requires binding to its receptor, using Klotho as co-factor [24, 25]. It is believed that tissue Klotho levels of the kidney decline with CKD and may thus be the mechanism behind FGF23 resistance [26–31]. Although FGF23 is thought to have effects also independently from Klotho [32], it is possible that elevated FGF23 concentrations reflect renal tissue Klotho deficiency, and that FGF23 resistance itself contributes to the increased relative risk regarding outcome. This indeed was suggested by Dominguez et al. [33] who used fractional excretion of phosphate (FePi) as a marker of FGF23 resistance, and observed that FePi modified the association of FGF23 with outcome in 872 patients with relatively mild CKD [mean estimated glomerular filtration rate (eGFR)71 ml/min/1.73 m^2^]. Those considered to be more FGF23 resistant had worse outcome. We questioned if the same could be observed in patients with more severe CKD, a situation associated with increased CV mortality, progression of CKD and higher FGF23 concentrations.

## Patients and methods

### Patients

We used baseline data of patients who participated in the MASTERPLAN (Multifactorial approach and superior treatment efficacy in renal patients with the aid of nurse practitioners) study [34]. MASTERPLAN was a randomized controlled clinical trial (ISRCTN73187232) performed in nine Dutch hospitals in which patients with CKD (eGFR 20–70 ml/min/1.73 m^2^) were randomized to receive either usual care by the nephrologist or intensified treatment with added nurse practitioner support. Inclusion started in April 2004 and ended in December 2005. In two centers, baseline 24 h urine was collected and samples were stored at −80 °C. Only patients from these centers were included in the current analysis as urinary analyses were required.

### Data collection

Details of the study methods are described elsewhere [34]. In brief, baseline measurements included a questionnaire recording smoking behavior and medication use. Physical examination consisted of measurement of height, weight and blood pressure. Laboratory assessment included fasting serum creatinine, calcium, phosphorus and PTH. Stored blood samples were used at a more recent date for measuring FGF23 using sandwich enzyme-linked immunosorbent assay (ELISA) (Immutopics San Clemente, CA, USA) measuring the c-terminal FGF23 [35]. The intra- and inter-assay coefficients of variation of this assay are <5 and <16 %, respectively [36]. Urine samples were thawed, acidified, and creatinine, calcium, and phosphate were measured using standard automated techniques. EGFR was calculated with the four-point Modification of diet in renal disease (MDRD) formula [37]. Fractional excretion of phosphate was calculated as (urine phosphate × serum creatinine)/(serum phosphate × urine creatinine). Clinical outcome in MASTERPLAN has been described elsewhere [38]. A composite end-point was defined consisting of the combination of death, CV events (myocardial infarction, cerebrovascular accident, percutaneous transluminal coronary angioplasty or coronary artery bypass graft) and renal failure (defined as need for renal replacement therapy, doubling of serum creatinine or death).

### FGF23 resistance

FGF23 increases phosphate excretion through reduced phosphate re-absorption. Thus, FGF23 resistance can be considered if a high FGF23 exists together with a low FePi.

### Statistical analysis

Baseline characteristics are reported as median values with interquartile range (IQR) for skewed data and as mean values with standard deviation (SD) for normally distributed data. Medians between groups were compared using the non-parametric independent samples median test. Natural logarithm transformation was applied for the skewed data. Spearman correlation coefficients were used to perform univariate analyses. Survival analyses were performed using Cox regression analyses in order to adjust for possible confounding by gender, age, systolic blood pressure, eGFR, PTH, proteinuria and smoking. In order to evaluate if FGF23 resistance expressed as FePi increased the effect of FGF23 on outcome, patients were categorized by FGF23 above and below the median combined with FePi above and below the mean into four categories. A product term for the categorized FGF23 and FePi was added again to Cox regression. In the presence of a positive interaction, the sum of hazard ratios (HR) for the combination of a high FGF23 and low FePi would be higher than the theoretical calculated HR of a high FGF23 and high FePi times the ratio of the HR of a low FGF23 and low FePi divided by the HR of the reference group. A second method to calculate the presence and direction of interaction was performed by calculating the relative excess risk due to interaction (RERI) [39, 40]. The confidence interval (CI) of the RERI was estimated using jackknife resampling. A positive additive interaction is present if the RERI is >0 and is statistically significant. Statistical significance was defined as a two sided *p* value of <0.05. Statistical analyses were performed using SPSS 20.0 (IBM SPSS software, IBM Corp, Armonk, NY, USA) and Stata 11.2 (StataCorp, College Station, TX, USA) software packages.

## Results

The two participating centers included 194 patients in the MASTERPLAN study. Urine samples were available for 166 patients. Baseline characteristics of these 166 patients grouped by FGF23 and FePi are shown in Table 1. The median age was 53 (IQR 45, 62) years, most patients were Caucasian and the median eGFR was 36 (IQR 28, 44) ml/min/1.73 m^2^. The median of proteinuria was 0.40 (IQR 0.20, 1.20) g/day, FePi 0.32 (IQR 0.25, 0.44) and FGF23 140 (IQR 81–236) RU/ml. People with higher FGF23 levels more frequently used vitamin D compounds.

**Table 1:** Baseline characteristics of patients grouped by FGF23 and FePi (*n* = 166)

Variable	Total group (*n* = 166)	Low FGF23, high FePi (*n* = 31)	Low FGF23, low FePi (*n* = 52)	High FGF23, high FePi (*n* = 51)	High FGF23, low FePi (*n* = 32)	*p* value
Gender, male, *n* (%)	112 (67)	26 (84)	28 (54)	40 (78)	18 (56)	0.01
Age, years (range)	53 (45–62)	52 (43–64)	53 (45–61)	54 (50–63)	49 (43–64)	0.38
BMI (kg/m^2^)	26 (24–29)	26 (23–27)	26 (23–28)	27 (24–30)	26 (23–31)	0.47
Race (Caucasian)	160	30	48	51	31	0.22
Cause of CKD						
Diabetic	14	1	4	7	2	0.62
Renovascular	9	1	3	4	1	
Glomerulonephritis	41	7	18	10	6	
Interstitial	29	8	6	8	7	
Congenital	8	3	3	0	2	
Cystic kidney disease	22	3	7	8	4	
Unknown	33	6	9	9	9	
Other	10	2	2	5	1	
Systolic BP (mmHg)	126 (118–139)	124 (116–134)	124 (116–135)	130 (119–144)	125 (118–136)	0.44
Diastolic BP (mmHg)	75 (70–81)	75 (71–78)	76 (70–81)	75 (70–83)	76 (68–83)	0.81
eGFR (MDRD4, ml/min/1.73 m^2^)	36 (27–44)	36 (29–45)	41 (36–52)	27 (20–34)	36 (27–42)	<0.01
Proteinuria (g/24 h)	0.40 (0.20–1.20)	0.40 (0.20–1.20)	0.30 (0.10-0.58)	0.80 (0.30–1.50)	0.60 (0.20–1.38)	0.01
Total cholesterol (mmol/l)	4.99 ± 0.98	5.10 (4.50–5.80)	5.00 (4.40–5.50)	4.80 (4.00–5.30)	5.15 (4.53–6.08)	0.18
Serum phosphate (mmol/l)	1.04 (0.92-1.20)	1.02 (0.94–1.13)	1.00 (0.89–1.09)	1.17 (0.96–1.40)	1.04 (0.90–1.20)	0.03
Serum calcium (mmol/l)	2.35 ± 0.15	2.37 (2.30–2.50)	2.38 (2.28–2.44)	2.34 (2.26–2.42)	2.33 (2.22–2.40)	0.49
Serum albumin (mmol/l)	40 ± 4	41 (40–43)	40 (38–42)	39 (37–42)	40 (37–41)	0.10
Serum PTH (pmol/l)	10.15 (6.68–16.18)	10.15 (8.18–16.73)	9.00 (4.65–12.00)	14.25 (9.00–23.23)	10.00 (6.08–15.85)	<0.01
Serum cFGF-23 (RU/ml)	140 (81–236)	78 (54–114)	81.25 (54.10–115.00)	278 (203–439)	204 (159–289)	<0.01
Fractional phosphate excretion	0.32 (0.25–0.44)	0.41 (0.36–0.46)	0.24 (0.19–0.28)	0.49 (0.40–0.54)	0.26 (0.22–0.29)	<0.01
Fractional calcium excretion	0.00 (0.00–0.01)	0.00 (0.00–0.01)	0.00 (0.00–0.01)	0.00 (0.00–0.01)	0.00 (0.00–0.01)	0.99
Vitamin D drugs (n, %)	33 (20)	3 (10)	5 (10)	18 (35)	7 (22)	0.004
Diabetes	35	2	8	18	7	0.01
Smokers	27	2	6	15	4	0.02
Events/PY	96/792	11/158	25/257	37/236	23/141	
Events/1000 PY	121	70	97	157	163	

Median with interquartile ranges (25–75 %) for skewed data

Mean with standard deviation (±SD) for normally distributed data

*FGF23* fibroblast growth factor 23, *FePi* fractional excretion of phosphate, *BMI* body mass index, *CKD* chronic kidney disease, *BP* blood pressure, *eGFR* estimated glomerular filtration rate, *MDRD4* 4-point modification of diet in renal disease, *PTH* parathormone, *PY* person years

### Correlations

Univariate analysis between FGF23 and parameters of kidney function and phosphate metabolism showed a significant inverse correlation between FGF23 and eGFR (*R* = −0.43, *p* < 0.01). We observed a significant positive correlation between FGF23 and serum phosphate (*R* = 0.29, *p* < 0.01), FePi (*R* = 0.36, *p* < 0.01), urine phosphate/creatinine ratio (*R* = 0.17, *p* = 0.03) and PTH (*R* = 0.30, *p* < 0.01). Of note, there was no significant correlation between FGF23 and serum calcium. In the multivariate analysis including sex, body mass index (BMI), age, PTH, eGFR, FePi, serum phosphate, serum calcium, total cholesterol, systolic blood pressure, proteinuria, diabetes and smoking, only eGFR and smoking were independent predictors of FGF23.

### FGF23 resistance

Clinical characteristics of the patients divided by the combination of FePi and FGF23 are reported in Table 1. Compared to patients with FGF23 concentrations below the median, the patients with higher concentrations had more proteinuria, a higher PTH, a higher phosphate and a lower eGFR. Patients with high FGF23 were also more frequently diabetics and more likely to smoke. Patients with a low FePi had lower serum phosphorus levels, a lower PTH, total cholesterol and were more likely female.

### Outcome

**Fig. 1:**
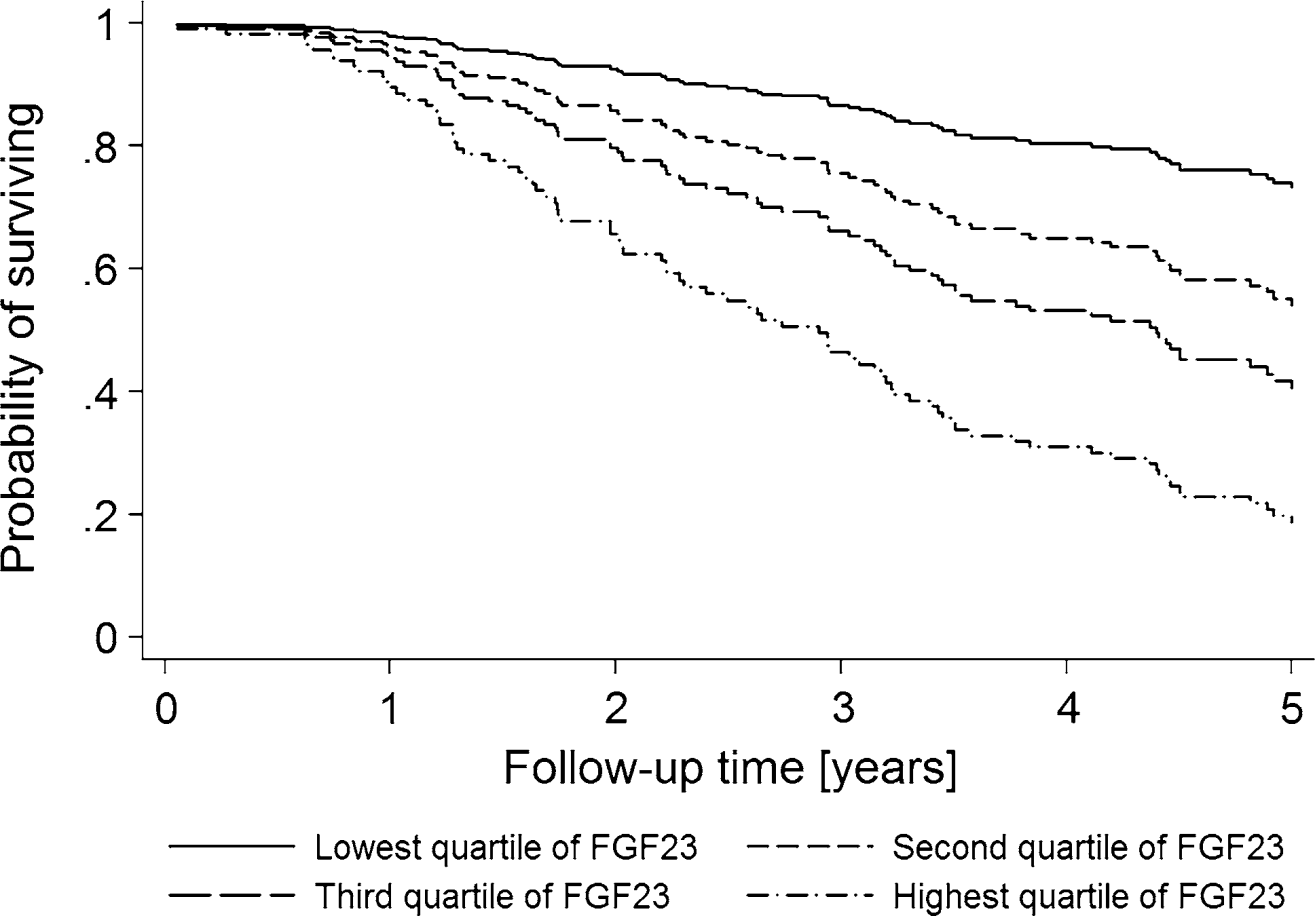
Cox curve for quartiles of FGF23 and the effect on composite outcome. Adjusted for age, sex, smoking, systolic blood pressure, proteinuria, eGFR, TmP/GFR, serum phosphate and PTH. FGF23, fibroblast growth factor 23; eGFR, estimated glomerular filtration rate; TmP/GFR, ratio of the maximum rate of tubular phosphate reabsorption to the glomerular filtration rate; PTH, parathormone

After a median of 4.8 years of follow up, 96 of the 166 patients (59 %) reached an endpoint defined as death (8/166), CV event (14/166), renal replacement therapy or doubling of creatinine (74/166). In the multivariate Cox regression analysis only lnFGF23, proteinuria and systolic blood pressure remained independent predictors of the composite outcome (Table 2). FePi was not associated with outcome. Moreover, the hazard ratio for LnFGF23 for outcome did not change after adjustment for FePi (Table 2). Figure 1 shows the Kaplan–Meier curve for the composite outcome according to quartiles of FGF23 after adjustment for baseline covariates.

**Table 2:** Univariate and multivariate Cox regression analysis for combined outcome

	Univariate			Multivariate		
	HR	CI	*p* value	HR	CI	*p* value
LN FGF23	2.17	1.65–2.84	<0.01	2.13	1.54–2.95	<0.001
FePi	3.39	0.86–13.47	0.082	0.23	0.04–1.52	0.13
Serum phosphate	3.57	1.72–7.45	<0.001	2.13	0.87–5.19	0.10
Proteinuria	1.27	1.15–1.41	<0.001	1.25	1.11–1.41	<0.001
MDRD	0.97	0.96–0.99	0.005	0.99	0.97–1.02	0.59
Age	0.99	0.97–1.00	0.158	0.99	0.97–1.00	0.11
Gender (male)	1.32	0.85–2.09	0.215	0.67	0.40–1.11	0.12
Smoking	1.75	1.08–2.84	0.022	0.98	0.54–1.75	0.93
Systolic blood pressure	1.02	1.00–1.04	0.001	1.02	1.00–1.03	0.02
PTH	1.02	1.00–1.03	0.017	1.00	0.98–1.02	0.98

*HR* hazard ratio, *CI* confidence interval, for other abbreviations, see Table 1

Tables 3 and 4 further show the hazard ratios for the associations between FGF23 and FePi categories and outcome. Cox regression, which was adjusted for age, sex, smoking, systolic blood pressure, proteinuria, eGFR and PTH, revealed a hazard ratio for the combination of high FGF23 and low FePi of 4.15 (95 % CI 1.85–9.30) on the combined outcome (Table 3). In the absence of hazard ratio modification, one would have expected the joint effect of a high FGF23 and low FePi to give a HR of 8.14 [=e^ln(2.56) + ln(3.18)^]. Likewise, the RERI was −0.60 (95 % CI −4.81 to 3.70). Similarly, the hazard ratio of a high FGF23 combined with low FePi on renal outcome was 3.44, lower than expected from the sum of both individual factors, HR = 7.77 [=e^ln(2.40) + ln(3.22)^].

**Table 3:** Multivariate cox regression on combined outcome for categories defined by FGF23 below and above the median combined with FePi below and above the mean

Combined survival	Participants	Events	HR	95 % CI
Low FGF23/high FePi	31	11	1	Reference
Low FGF23/low FePi	52	25	2.56	1.13–5.80
High FGF23/high FePi	51	37	3.18	1.47–6.87
High FGF23/low FePi	32	23	4.15	1.85–9.30

This model included FGF23 and FePi as categorical variables and their product term

The model was adjusted for factors shown and age, sex, smoking, systolic blood pressure, proteinuria, eGFR, PTH

Hazard ratio product for the product term is 0.51 (95 % CI 0.19–1.34), *p* = 0.17

RERI is −0.60 (95 % CI −4.81 to 3.70), *p* = 0.79

*RERI*, relative excess risk due to interaction, for other abbreviations, see previous tables

**Table 4:** Multivariate cox regression on renal outcome for categories defined by FGF23 below and above the median combined with FePi below and above the mean

Renal survival	Participants	Events	HR	95 % CI
Low FGF23/high FePi	31	9	1	Reference
Low FGF23/low FePi	52	20	2.40	0.81–6.34
High FGF23/high FePi	51	34	3.22	1.31–7.84
High FGF23/low FePi	32	18	3.44	1.37–8.62

This model included FGF23 and FePi as categorical variables and their product term

The model was adjusted for factors shown and age, sex, smoking, systolic blood pressure, proteinuria, eGFR, and PTH

Hazard ratio product for the product term is 0.44 (95 % CI 0.14–1.41)

RERI is −1.18 (95 % CI −8.10 to 5.73), *p* = 0.74

For all abbreviations, see previous tables

## Discussion

In this study we tested the hypothesis that FGF23 resistance may contribute to the increased risk for morbidity and mortality associated with elevated FGF23 levels in patients with advanced CKD. This hypothesis was based on a recent study showing interaction between FePi and FGF23 levels on outcome in patients with mild CKD [33]. Our study showed that there was no interaction between FGF23 levels and FePi on this outcome, and thus we could not confirm this hypothesis in subjects with more advanced CKD and higher FGF23. This suggests that in patients with advanced CKD, FGF23 itself and not FGF23 resistance determines the risk for adverse outcome.

This study confirmed that FGF23 is an independent predictor of outcome. Moreover, multivariable adjustments that included FePi did not mitigate the HR of FGF23. FGF23 concentrations increase during progression of CKD and several studies have shown that FGF23 is associated with mortality in hemodialysis patients as well in patients with CKD [16, 17, 41–43]. Also in our analysis FGF23 remained a predictor of outcome after adjustment of eGFR. Interestingly, the HR of eGFR itself on the composite outcome that included progression of CKD was lost in the multivariable model. This suggests that the well-established risk of CKD may actually be accounted for by high levels of FGF23 that accompany CKD. Obviously, increased FGF23 levels might merely reflect the severity of other unmeasured risk factors. Cohort studies, for instance, have reported associations of FGF23 with left ventricular hypertrophy [44, 45], progression of kidney failure [16, 19, 20], and with several cardiovascular risk factors, such as endothelial dysfunction and arterial stiffness, in the general population as well as in early CKD, in the absence of clinically evident disturbances in phosphate metabolism [21, 46, 47].

Our findings appear to contrast with the report by Dominguez et al. [33] who concluded that the association of FGF23 with outcome was reinforced if high FGF23 was accompanied by a low FePi, suggesting that kidney FGF23 resistance modifies the association between FGF23 and outcome. There are several possible explanations for this discrepancy. The most important factor is the obvious difference in patient population and outcome. Dominguez et al. studied patients that participated in the Heart and Soul study, which included patients with prevalent occlusive coronary artery disease and normal to slightly decreased eGFR, mainly attributed to vascular disease. Importantly, in the eGFR range of the Heart and Soul study (stage II CKD), CKD is not an important contributor to overall risk [1]. In contrast, our patients had moderate-severe CKD and often defined kidney disease whereby outcome was mainly determined by renal failure. The second explanation could be the difference in methodology. We performed interaction analyses by calculating the direction of the interaction using the low FGF23 and high FePi as reference group. Dominguez documented the p-value though the direction seems opposite and used low FGF23 and low FePi as reference group.

Another study examined the association of a poor phosphaturic response to FGF23, as a sign of FGF23 resistance, with abdominal aortic calcification in CKD stages 3–4 [48]. In this study, FGF23 correlated well to FePi except in the group of patients with severe aortic calcification, while eGFR and PTH correlated well to FePi irrespective of the amount of calcification, suggesting an association between FGF23 resistance and severe aortic calcification. A shortcoming of this study however is that the ratio of FePi to FGF23 (FePi/FGF23) was used as a marker of resistance where this ratio was mostly determined by the denominator FGF23 as there was no difference in FePi between groups. Therefore, outcome on calcification score was mainly determined by FGF23. Overall, we cannot exclude the possibility that FePi values might indeed modify FGF23-related risk in patients with early-stage CKD. In patients with more severe CKD, however, the increased risk is predominantly determined by the higher FGF23 levels, and not by kidney resistance of its effects. Furthermore, FePi was not associated with outcome at either univariate or multivariate analysis. Previous studies showed that also FePi was not associated with mortality and cardiovascular events in a model adjusted for FGF23 and eGFR [17, 33, 49]. This might be because in CKD other (non-defined) risk factors might outweigh the risk of tubular resistance to FGF23, or that more advanced tubular damage inhibits phosphate reabsorption by other mechanisms than the physiological effects of FGF23, as can be observed, for instance, in Fanconi’s syndrome.

Our results thus refute a significant contribution of FGF23-resistance to outcome in patients with CKD 3-4. In addition to the arguments described above, it should, however, be borne in mind that there lacks a good representative parameter and validated measure of FGF23-resistance, in that both TmP/GFR (ratio of the maximum rate of tubular phosphate reabsorption to the glomerular filtration rate) and FePi might not reflect FGF23-resistance optimally, especially in the setting of more advanced CKD.

Our study has some limitations, all being consequences of the post hoc nature of the current analysis. The main limitation is the relatively small number of patients included. Although the event rate was high, the events were mainly in the group of patients with highest FGF23 levels. Especially for the interaction analysis that we performed, a larger number of patients would have been preferable. Due to the small number of patients, we used a composite end-point. Our study had a low power to evaluate single end-points with adjustment for competing risks. Another limitation is that the study is a post hoc analysis of mostly Caucasian patients with more severe CKD and results might not be directly applicable to other populations. Furthermore, although serum phosphate was measured in a fasting state, FePi was not a timed specimen before blood sample collection but was calculated from 24 h urine samples. This might have led to higher FePi values, and an underestimation of FGF23 resistance because fasting phosphate concentrations are generally lower than the average daytime value. A final limitation is that we used a composite end-point, and that in larger studies outcomes may differ for individual components of the currently used composite end-points.

Strengths of our data are the prospectively collected data, and the inclusion primarily of patients in a stage of CKD where CKD has proven impact on clinical outcome.

## Conclusion

In conclusion, in this study in patients with a median eGFR of 36 (IQR 27–44) ml/min/1.73 m^2^, FePi did not modify the association of FGF23 with outcome. Therefore, in advanced CKD, the role of FGF23 resistance expressed by FePi is negligible compared to the risk predicted by increased concentrations of FGF23 itself.
